# Prevention of New Respiratory Episodes in Children with Recurrent Respiratory Infections: An Expert Consensus Statement from the World Association of Infectious Diseases and Immunological Disorders (WAidid)

**DOI:** 10.3390/microorganisms8111810

**Published:** 2020-11-17

**Authors:** Susanna Esposito, Marcus Herbert Jones, Wojciech Feleszko, José A. Ortega Martell, Oana Falup-Pecurariu, Natalia Geppe, Federico Martinón-Torres, Kun-Ling Shen, Michael Roth, Nicola Principi

**Affiliations:** 1 Pediatric Clinic, Pietro Barilla Children’s Hospital, University of Parma, 43126 Parma, Italy; 2 School of Medicine, Pontifical Catholic University of Rio Grande do Sul (PUCRS), Porto Alegre (RS) 90619-900, Brazil; mhjones@pucrs.br; 3 Department of Pediatric Pneumonology and Allergy, The Medical University of Warsaw, 00-927 Warsaw, Poland; wojciech.feleszko@wum.edu.pl; 4 Department of Immunology, Universidad Autónoma del Estado de Hidalgo, Pachuca, Hidalgo 42082, Mexico; drortegamartell@prodigy.net.mx; 5 Faculty of Medicine, Transilvania University, Children’s Clinic Hospital, 500036 Brasov, Romania; oanafp@yahoo.co.uk; 6 Department of Paediatrics, Sechenov First Moscow State Medical University, 115093 Moscow, Russia; geppe@mail.ru; 7 Translational Pediatrics and Infectious Diseases, Department of Pediatrics, Hospital Clínico Universitario de Santiago de Compostela, 15706 Santiago de Compostela, Spain; federico.martinon.torres@sergas.es; 8 China National Clinical Research Center for Respiratory Diseases, Department of Respiratory Medicine, Capital Medical University, National Center for Children’s Health, Beijing 100045, China; kunlingshen1717@163.com; 9 Pulmonary Cell Research and Pneumology, Department of Biomedicine and Internal Medicine, University Hospital Basel, 4002 Basel, Switzerland; michael.roth@usb.ch; 10 Università degli Studi di Milano, 20122 Milan, Italy; nicola.principi@unimi.it

**Keywords:** bacterial lysates, pidotimod, probiotics, respiratory infection, respiratory recurrences, vitamin

## Abstract

In healthy infants and young children, the development of respiratory tract infections (RTIs) is extremely common. In this paper, we present an international consensus of the available approaches for the prevention of recurrent RTIs in children, including the atopic/allergic ones as well as those with asthma. Few convincing measures for reducing the frequency and clinical relevance of recurrent respiratory episodes in RTI-prone children have been developed until now. Among the most recently suggested measures, immunotherapy is attractive, but only for OM-85 is there a sufficient number of well-conducted clinical trials confirming efficacy in RTIs prevention with an adequate safety profile. In the case of probiotics, it is not clear which bacteria can offer the best results and which dosage and schedule of administration are the most effective. The problems of dosage and the schedule of administration are not solved also for vitamin D, despite some promising efficacy results. While we wait for new knowledge, the elimination or reduction as much as possible of the environmental factors that favor RTIs, vaccination when available and/or indicated, and the systematic application of the traditional methods for infection prevention, such as hand washing, remain the best measures to prevent recurrent infections in RTI-prone children.

## 1. Background

In otherwise healthy infants and young children, the development of respiratory tract infections (RTIs) is widespread. It has been demonstrated that in the first 6 years of life, a child without any underlying pathological condition experiences several RTI episodes that, although generally mild and spontaneously solving, significantly impact the quality of life of the patient and cause relevant problems to the family, society, and health care system [1,2,3]. When recurrences are very frequent, the child is defined as prone to RTIs, and measures to reduce the frequency, clinical relevance, and socioeconomic impact of RTIs are usually requested by parents and planned by the pediatrician in charge [4].

Prevention of recurrent RTIs in otherwise healthy children has recently become the focus of several clinical studies. Despite the benefits in reducing the burden of RTIs with some of these interventions, we have only partial success so far. There is no consensus on which children should be considered RTI-prone (i.e., suffering from recurrent RTIs), as different countries use different criteria. The specific airway infection sites, the total number of RTIs, and the period of life during which infections should occur vary from country to country. For example, in China, a child is considered to be RTI-prone if he/she suffers from ≥7 episodes/year, ≥6/year, and ≥5/year of upper RTI at 02 years, >25 years, and >5–14 years, respectively [5]. When lower RTIs occur, the number of infections needed to be considered RTI-prone is reduced to 2–3 episodes/year. In the USA, the definition includes the site of infection, and age is limited to 6 years [6,7,8]. For acute otitis media (AOM), at least three episodes within 6 months or four or more episodes within 12 months are the criteria to define a child otitis-media-prone [3]. Recurrent infectious rhinitis is usually defined as more than five episodes per year, and recurrent pharyngitis or tonsillitis is defined as more than three episodes within 12 months [3]. Further diversifications are made in Central and South America as well as in Europe, including Italy, where, to be identified as RTI-prone, children should suffer from ≥6 RTIs per year or ≥1 upper RTIs per month from September to April or ≥3 lower RTIs per year [3,9]. A reduction of risk factors (i.e., exposure to passive smoking, use of a pacifier, day-care attendance) represents the first strategy against respiratory recurrences, but it does not always work. Vaccine or drug prophylaxis of RTIs is limited [10]. Most infectious episodes that occur in children with recurrent RTIs are of viral origin, and bacterial vaccines and antibiotics have no preventive role [11]. Several viruses play a role in the causes of RTIs [12], but vaccines and drugs are currently available only against influenza viruses. Although several factors that can favor RTIs have been identified [13,14,15] and also microbiome seems to have a role [16], the chances of reducing their weight in the determination of recurrences are very small or none. No data are available regarding possible genetic modifications that increase the risk of infections [17]. No definitive solution can be suggested regarding environmental factors such as pollution, day-care attendance, passive smoking, or the lack of prolonged breastfeeding [16].

In recent years, based on evidence that in the first years of life the immune system is immature and partially unable to defend against infectious agents [18], the use of immunotherapy, i.e., measures that are able to stimulate and/or regulate the activity of some components of the immune system, thereby making host defenses more efficient, has been advocated [19,20]. Moreover, attempts to use vitamins [21,22,23], probiotics [24,25,26,27], oligo-elements [27], and complementary and alternative medicine [28,29] have been made. Unfortunately, the real benefits offered by some treatments are dubious, and they should be avoided until supported by evidence.

In this paper, we present an international consensus of the available approaches for the prevention of RTIs in children, including the atopic/allergic ones as well as those with asthma. We hope it will assist pediatricians and general practitioners in making better decisions when attending children with recurrent RTIs. A search in Pubmed for all of the studies published up to April 2020 was performed using “children”/“paediatric”/“pediatric” and “respiratory infection” and “prevention” as keywords. The search was limited to articles published in English that provided evidence-based data.

## 2. Immunotherapy

During the last 50 years, several immunomodulating preparations have been developed. Some of them, such as those containing transfer factors [30], isoprinosine [31], and thymus hormones [32], after some attempts to evaluate their efficacy in the prevention of recurrent RTIs of children, have been abandoned and presently are no longer considered in this regard. Other preparations, such as pidotimod (PDT) and bacterial lysates, developed more than 30 years ago, have recently attracted new interest and have been the object of numerous studies. Finally, biologically active polysaccharides, known since the 1960s and initially considered dietary supplements, are presently studied as real drugs to be used even in the prevention of recurrent RTIs.

### 2.1. Pidotimod

PDT is a synthetic dipeptide molecule (3-l-pyroglutamyl-l-thiazolidine-4-carboxilic acid) that was introduced in Italy in 1993 and later in some other European countries (Russia, Ukraine, and Greece), China, Mexico, and other countries of Central and South America. It is not licensed in most European countries or North America. In vitro and in vivo studies, both in experimental animals and in humans, have shown that PDT can positively influence innate and adaptive immunity [33]. The administration of PDT is associated with the increased expression of Toll-like receptor (TLR) 2 and HLA-DR molecules, the induction of dendritic cell maturation, the release of pro-inflammatory mediators [34,35], the stimulation of T lymphocyte proliferation with polarization toward a Th1 phenotype, and the suppression of Th2 cytokines [36]. Moreover, PDT increases the cytotoxic activity of natural killer cells and the phagocytosis of neutrophils. Finally, although direct evidence of activity on B cells is lacking and PDT administration is not associated with a significant increase in serum antibody titers, an increased production of secretory IgA has been reported [37]. 

PDT has been found to be well-tolerated and have a good safety profile, and all these findings have suggested a potential role of this drug in the prevention of recurrent RTIs in children provided that its pharmacokinetic and pharmacodynamic characteristics are observed. PDT is given orally, has a half-life of approximately 4 h, and is eliminated unchanged by the kidney. When given on an empty stomach, PDT has a bioavailability of 42–44%, but absorption is strongly influenced by food, with a reduction of oral bioavailability up to approximately 50%. This indicates that to optimize absorption, PDT should be given at least 2 h before or 2 h after meals. Moreover, PDT is licensed for children ≥3 years of age, and this is a partial limitation because the frequency of recurrent RTIs is extremely high in the first three years of life. Although different dosages have been prescribed in China, PDT is generally administered at a dose of 400 mg one or two times a day. The duration of the effect of PDT is not precisely defined, although continuous daily administration for 1–3 months has been used in most studies [33]. However, in some cases, administration for 10 days each month for 3-6 months was also used [38]. 

The results of clinical trials that specifically planned to evaluate PDT for the prevention of recurrent RTIs in children seemed to confirm the potential efficacy of the drug and its safety [39,40,41,42,43,44,45,46,47,48,49]. However, the analysis of these studies raises doubts about the reliability of the results and related conclusions. The first studies carried out in Italy in the early 1990s generally had significant methodological limitations and cannot provide reliable results [39,40,41,42,43,44,45], while more satisfactory conclusions can be drawn from the analysis of the most recent studies. A meta-analysis of 29 randomized clinical trials published up until February 2018 [46], including 22 new clinical trials carried out from 2002, revealed that compared to controls, children with a history of recurrent RTIs receiving PDT had a significantly lower risk of new episodes of infection (relative risk (RR) 1.59, 95% (CI) 1.45–1.74, *p* < 0.00001) during the study period. The effect was evidenced during the period of drug administration (RR 1.72, 95% CI 1.47–2.02, *p* < 0.00001) but was maintained even some months after the end of treatment. Seven to twelve months after beginning prophylactic treatment, the risk of ≥2 RTIs was significantly higher among controls than among PDT-treated patients (RR 1.44, 95% CI 1.31-1.58, *p* < 0.00001).

Moreover, PDT administration was associated with a lower antibiotic use and, in subjects who developed RTI, with a significant reduction in the duration and severity of the signs and symptoms of disease [46]. However, even these favorable results are strongly questioned by the existence of serious limitations of the studies included in this meta-analysis. Among the 22 new studies, only three [47,48,49] were carried out in Europe and published in peer-reviewed journals with at least the abstract in English. All the other new studies were in Chinese and in most cases were published in journals without peer review. Limitations of the studies included in the meta-analysis are clearly highlighted by the same authors [46], who reported that most of the included trials had poor methodological quality, such as lack of sufficient information on allocation concealment, lack of sufficient details on the randomization method, and using a non-blind method. Moreover, studies were significantly heterogeneous. The dosages of PDT and the schedule of administration were substantially different among studies, the baseline clinical characteristics of the enrolled children were frequently not precisely defined, and the type and severity of previous RTIs were not detailed. 

All these findings explain why results on PDT efficacy in RTI prevention must be interpreted with caution and that further studies are needed before PDT can be included among the measures to use for the prevention of new episodes of RTI in RTI-prone children. 

### 2.2. Bacterial Lysates

#### 2.2.1. OM-85

OM-85 is a lysate of 21 strains of bacterial pathogens derived from the eight major species and sub-species that are a common cause of RTIs (Haemophilus influenzae, Streptococcus pneumoniae, Klebsiella pneumoniae, Klebsiella ozaenae, Staphylococcus aureus, Streptococcus pyogenes Streptococcus viridans, Moraxella catarrhalis) [19]. Several studies have shown that both innate and adaptive immunity are strongly influenced by OM-85 [50,51,52,53,54]. Oral OM-85 administration stimulates dendritic cell maturation in gastrointestinal Peyer’s patches, resulting in the increase of the immune defenses also in the lung mucosa [19]. It favors T lymphocyte proliferation with the upregulation of the Th1-specific cytokine interferon-γ and the reduction of Th2-specific interleukin (IL)-4. Consequently, the Th1/Th2 imbalance, which is typical of the first periods of life, is corrected. Moreover, OM-85 stimulates antimicrobial peptide release and the activation of macrophages with the increased secretion of antiviral cytokines and chemokines such as IFN alpha and beta. Finally, B cell-activating cytokines are produced with increases in both serum and mucosal polyclonal immunoglobulins (IgG and IgA) [50,51,52,53,54].

As well as defending against viral and bacterial infections, OM-85 treatment was shown to control inflammation to reduce tissue damage, thus providing a two-stage process of combat and control. In this regard, in experimental animals, it has also been shown to downregulate the immune system in airway chronic inflammatory states, such as chronic rhinosinusitis [55,56], by decreasing the levels of pro-inflammatory cytokines (e.g., IL-1β) in a dose-dependent manner [57], dampening recruitment of inflammatory cells [50], and increasing the levels of anti-inflammatory cytokines (e.g., IL-10) [58], thus reducing tissue damage. Other effects of OM-85 include increasing tolerogenic dendritic cells (CD103+); the activation of T cells with conversion to Treg cells; and decreasing Type 2 DCs [19].

OM-85 may also aid the maturation of the immune system in children by correcting Th1/Th2 imbalance through increasing Th1 cytokines (IFN-γ), increasing Treg cytokines (IL-10), and decreasing Th2 cytokines (IL 4, IL 5, IL 13) [57]. The correction of this Th2-oriented imbalance and other anti-inflammatory activity (such as decreasing inflammatory cell infiltration, and decreasing eosinophils, neutrophils, macrophages, and T and B cells) may help to reduce atopic responses related to wheezing and asthma [19]. For example, increased levels of IL-10 are associated with decreased airway inflammation, subsequent tissue remodeling, and decreased hypersecretion. These effects combined with the reduced risk of recurrent RTIs, which predispose to asthma and cause exacerbations, form the mechanistic framework for a reduced risk of these conditions [59]. In addition, OM-85 has recently shown several immunoregulatory properties that are of relevance in atopic children prone to bronchial hyperactivity. The beneficial effects of OM-89 in atopy might be related to the potential ability to mimic the functionality of the host–microbiota [19]. 

Commercial products containing OM-85 are licensed in several European countries (Austria, Belgium, Bulgaria, Czech Republic, Germany, Greece, Hungary, Italy, Latvia, Lithuania, Luxembourg, Malta, Poland, Portugal, Romania, Slovakia, and Slovenia) and in several Asian, Central, and South American countries. In North America, a large clinical trial sponsored by the National Institute of Health (NIH) is ongoing in otherwise healthy children to evaluate protection from RTIs offered by OM-85. The lysate is given orally, starting from the age of 6–12 months, generally at a dosage of 3.5 mg once a day for 10 days for 3 consecutive months starting from the beginning of autumn to have the highest immune protection in the winter season when the risk of RTIs is higher.

Several clinical trials have evaluated whether immune modulation derived from OM-85 administration could reduce the risk of new infectious episodes in children with recurrent RTIs. The results were generally in favor of OM-85 versus placebo or control group. Fifty-three studies were included in a meta-analysis, with a total of 2491 children receiving OM-85 and 2360 controls [53]. A significant reduction in the frequency of new RTIs was evidenced in the group of treated children compared to controls (mean difference (MD) −2.33, 95% CI −2.75 to −1.90, *p* < 0.00001). Moreover, prophylaxis was associated with a shorter therapeutic time of antibiotic treatment (MD −4.10 days, 95% CI −4.52 to −3.67, *p* < 0.00001) and with a lower duration of infection (MD −3.13 days, 95% CI −3.91 to −2.35, *p* < 0.00001), febrile time (MD −2.91 days, 95% CI −3.75 to −2.07, *p* < 0.00001), cough (MD −5.26 days, 95% CI −6.4 to −4.12, *p* < 0.00001), and wheezing (MD −3.37 days, 95% CI −4.52 to −2.22, *p* < 0.00001) compared to controls. However, when the characteristics of the studies were analyzed, it was evidenced that most of them had significant methodological problems, only 11 were written in English [58,60,61,62,63,64,65,66,67,68,69], and they were published in peer-reviewed journals.

Moreover, the size of the studies was generally small, with only two studies among those published in English with at least 100 children in each intervention group [62,64,70]. Power calculations were generally not performed making conclusions highly debatable. Finally, the methodology was not clearly described in most of the studies. Only 14 and seven studies reported the correct randomization method and the right allocation method, respectively, and only 12 were double-blinded [64]. However, better conclusions can be drawn from the meta-analysis of the studies whose methodological characteristics can be considered acceptable. Schaad et al. [71] analyzed the studies published up until 2007, excluding all those that were unblinded or incomplete and including only those that had enrolled children with a documented history of recurrent RTIs (defined as at least three episodes of upper respiratory tract infections during the last 12 months). Eight studies were selected [58,61,62,63,64,65,66,67], and among them, six [49,50,51,52,53,60,61,62,63,64,65,66,67,68,69,70,71,72,73,74,75,76] were considered homogeneous. When these studies were pooled, it was calculated that children (mean age 6.27 ± 3.60 years) who had received OM-85 10 days each month for 3 consecutive months and were followed for a total of 6 months had in this period a mean number of RTIs that was significantly lower (−1.10; 95% CI −1.64 to −0.56) that in the placebo-receiving controls (mean age 6.41 ± 3.57 years). Moreover, it was evidenced that the effect of OM-85 was more significant for younger children and those with a higher number of RTIs within the previous year. Although demographic and clinical characteristics of children enrolled in these studies were in some cases not uniform, this analysis seems to indicate an actual possibility for the use of OM-85 to prevent new recurrences of RTI in RTI-prone children. Another meta-analysis performed by Del Rio Navarro and the Cochrane group showed a reduction of the mean rate of acute RTIs of 36% in children with recurrent RTIs treated with OM-85 [62]. This conclusion seems further supported by the results of a recent randomized, double-blind, placebo-controlled study. Esposito et al. enrolled 288 children aged 1 to 6 years with recurrent RTIs who were randomized in a 3:3:1 ratio to receive OM-85 for 3 months, placebo for 3 months, or OM-85 for 6 months [72]. The emergence of RTIs was monitored for 6 months, starting from the first OM-85 or placebo administration. The number of RTIs and of children who suffered from at least one RTI was significantly lower in the group receiving OM-85 for 3 months than receiving placebo (33% vs. 65%, *p* < 0.0001). In addition, the mean number of days of absence from day-care for children (4.49 vs. 5.10, *p* = 0.04) and the number of working days lost by parents were significantly lower in children treated for 3 months compared to those receiving placebo and those receiving OM-85 for 6 months (1.76 vs. 2.58, *p* = 0.004). Interestingly, in this studied population, no difference in RTI incidence was observed between children given OM-85 for 3 months and 6 months, suggesting that the protection induced by a 3-month prophylactic treatment is maintained for some months after the end of treatment, therefore covering the entire period at highest risk of recurrent RTI. As the third arm was only exploratory, this conclusion should be made carefully. However, a study by Razi et al. showed the long-lasting effect of a 3-month treatment with OM-85 [61]. Moreover, in another recent study, OM-85 was shown to be effective even when administered in successive years without any increase in adverse event incidence [72]. As the risk of recurrent RTIs in RTI-prone children generally lasts several years, this finding seems to indicate that OM-85 could be a measure to protect children for the entire at-risk period, generally the pre-school period. 

Overall, on the available evidence and the clinical practice, these data confirm the efficacy and safety of OM-85 for the prevention of respiratory recurrences.

#### 2.2.2. Ribomunyl, PBML, and LW50020

Ribomunyl is an immunostimulant that was licensed in France in the 1980s and later in a large number of countries, although the product is not available on the market worldwide anymore. It is composed of proteoglycans from Klebsiella pneumoniae and ribosomes from four of the most commonly encountered bacterial strains in respiratory tract infections (Klebsiella pneumoniae, Streptococcus pneumoniae, Streptococcus pyogenes, and Haemophilus influenzae) [74]. Ribomunyl increases the expression of adhesion molecules on phagocytic cells; it favors the maturation of dendritic cells, triggering a Th1 response; it improves innate and adaptive cytokine release; and it induces a relevant increase in antibody production, most of the IgA isotype [43]. Substantially, the biological effects of Ribomunyl were quite similar to those evidenced by OM-85. 

Some studies have evaluated the effect of Ribomunyl administration on children with recurrent RTIs, showing, as reported by some meta-analyses and systematic reviews, that this immunostimulant can reduce both the number of new respiratory infections and the number of antibiotic courses compared to placebo in children with recurrent RTIs [75,76]. However, there are few studies, and they enrolled a relatively low number of children. Moreover, most of them are dated, and in most cases, they have relevant methodological limitations. Consequently, no definitive conclusions about the use of Ribomunyl for the prophylactic treatment of children with recurrent RTIs can be drawn.

Even fewer data have been collected for two other lysates, PBML [77] and LW50020 [78] with the absence of new studies. Both are orally delivered and can stimulate immune defenses. However, clinical evaluation was very limited, and no conclusive data in children with recurrent RTIs have been collected to support their use.

## 3. Biologically Active Polysaccharides

Several carbohydrate polymers that can be obtained from fungi, yeast, bacteria, algae, and plants have biological effects, including immunomodulation, which can derive from a direct effect or from the induction of complex reaction cascades. B-glucans are the best studied because of their pluripotent biological properties with an enhancement of the activity of both innate and adaptive immunity [79]. B-glucans have been used for oral treatment and the prevention of RTIs in subjects of any age with contrasting results in terms of efficacy but with substantial evidence for safety and tolerability [80]. Regarding recurrent RTIs and children, most of the available data have been collected using pleuran—insoluble β-glucans from Pleurotus ostreatus, a mushroom—given in a syrup. Even in these subjects, the real role of B-glucans for prevention of RTIs remains debatable. When pleuran was administered to pre-school-age children every day for 3 months before the winter season, a reduction in the incidence of RTIs compared to the previous year was demonstrated [81,82,83]. Moreover, the potential preventive effects of pleuran supplementation on respiratory recurrences were suggested by a double-blind, placebo-controlled, multicenter randomized trial carried out in a group of 175 children aged 5.65 years [84]. The children received the syrup containing the medication or a placebo for 6 months and were followed up for 6 additional months. Treatment was associated with a significant reduction in respiratory morbidity, as, during the study period, 36% and 21% of the children in the active and placebo groups, respectively, did not suffer from any RTI (*p* < 0.05). Significantly reduced numbers of influenza and flu-like diseases and lower RTIs were also observed in the treatment group than in the placebo group (0.20 ± 0.55 vs. 0.42 ± 0.78 per 12 months, *p* < 0.05). Finally, contrary to placebo, pleuran-insoluble β-glucan administration resulted in a significant increase in IgG, IgA, and IgM isotypes, in an increase in the number of NK cells, and a relevant reduction in the slowdown speed of T-cytotoxic lymphocytes. However, a global evaluation of these and the other studies seem to indicate that, in most cases, the published studies had several weaknesses that should be resolved and addressed in further clinical trials and research. Only one randomized, double-blind, placebo-controlled trial measured the impact of β-glucans on children with recurrent RTIs. Additionally, the number of enrolled subjects was small in several studies; the criteria used to select patients for enrollment could be debated; and the optimal dose, duration, and timing of β-glucan application have not been clearly defined. The biologically active polysaccharides that offer the most exceptional prophylactic efficiency have not been established. Finally, the problem of the standardization of the production and extraction procedures to achieve the highest purity of the active substance from the natural β-glucan sources has not been solved. Equally unsolved is the problem of the possible combination of different β-glucans or the combination of a β-glucan with other plant-produced substances, such as resveratrol, to which immunostimulant activity could be ascribed [85]. The only study performed in children with recurrent RTI in which the prophylactic impact of carboxymethyl-β-glucan plus resveratrol on the incidence of new respiratory episodes was tested is debatable mainly for a number of methodological limitations [86].

## 4. Probiotics

According to the FAO/WHO definition, probiotics are live microorganisms which, when administered in adequate amounts, confer a health benefit on the host [87]. Gut and respiratory tract dysbiosis is observed in several diseases [88,89,90,91,92,93]. Probiotics appear to restore, at least in part, healthy microbiota in the gut and respiratory tract and reduce the clinical manifestations of diseases. This is because probiotics can explain several beneficial actions among which a relevant role is played by the influence on the immune system with an increase in host defenses. The production of antimicrobials is increased [94], toxin activity is blocked [95], pathogenic bacteria grow is contrasted [96], and immune system functions are improved with the enhancement of humoral and cellular immunity [97]. Starting from these premises, the use of probiotics, mainly Lactobacillus spp. and Bifidobacterium spp., to prevent and treat RTIs has been repeatedly suggested. Unfortunately, most of the studies regarding the prevention of RTIs in pediatrics were carried out in otherwise healthy children. Consequently, data on the effect of probiotics for the prevention of new respiratory episodes in RTI-prone children are very few. In a clinical study [98], the effect of Bifidobacteria administered for 2 months was assessed. The probiotic dosage varied according to the age of the children. During the year following the start of the trial, the average frequency of acute RTIs, the average duration of cough and fever, and the number of antibiotic prescriptions were all significantly lower (*p* < 0.05) in treated children than in untreated controls. Positive results were also reported in the prevention of recurrent AOM. Children who received oral Lactobacillus salivarius PS7 daily for 6 months during this period suffered from a significantly lower number of AOMs (*p* < 0.05) than during the 6-month period before the probiotic intervention [99]. However, a Cochrane review was less optimistic as, after the evaluation of five trials, it concluded that in AOM-prone children, probiotics were not effective, as the risk of new episodes was quite similar in children receiving probiotics and in those given placebo (RR 0.97, 95% CI 0.85–1.11) [100]. 

The possibility of the prevention of recurrent AOM using a local administration of probiotics is also unclear. The example of Streptococcus salivarius 24SMB is paradigmatic in this regard [101]. The number of children who did not experience any AOM was higher in children who were given a probiotic nasal spray than among those in the placebo group (30.0 vs. 14.9%; *p* = 0.076); however, this difference was not significant. When the analysis was limited to children who were colonized by the sprayed probiotic, the difference became statistically significant (*p* = 0.03). 

These studies have important limitations that deserve mention. The total number of enrolled children was always very low, and in most studies, the methods were highly questionable, and the results were debatable. Moreover, the heterogeneity of studies regarding the type of probiotic, dose, and duration of administration was very high. All of these factors lead to the conclusion that presently, it is not possible to establish whether recurrences of RTIs can be prevented by probiotics and, if prevention is possible, which probiotics, in what dosage, and in what scheme of administration can be truly effective.

Moreover, relatively little support for the hypothesis that probiotics can be effective in children with recurrent RTIs derives from the analysis of studies that have generically assessed the impact of probiotics on the incidence of RTIs in the pediatric population, even if without previous recurrences. Although most of these studies seem to indicate that probiotics are effective in this regard, a more in-depth analysis leads to less optimistic conclusions, highlighting the need for further studies. The available data do not provide answers to all of the questions that arise when probiotics have to be used to prevent RTIs. All these problems are evidenced by the results of a meta-analysis that do not allow us to recommend probiotics as a real solution for RTI prevention. In a meta-analysis of studies published up until May 2011 including both adults and children, it was found that probiotics were more beneficial than placebo in the prevention of upper RTIs and that their use was associated with reduced antibiotic consumption [102]. In particular, the number of participants with at least one episode of upper RTI was significantly lower among the individuals receiving probiotics than among controls (odds ratio (OR) 0.58; 95% CI 0.36–0.92). The effect was even more evident when the development of at least three episodes in the study period was considered (OR 0.53; 95% CI 0.36–0.80). Since the results of the pediatric studies were similar, it was suggested that probiotics could be effective for the prevention of RTIs in children with several episodes of RTIs. However, this conclusion was questioned, as the meta-analysis included trials with relevant heterogenicity due to differences in inclusion and exclusion criteria, primary and secondary outcomes, diagnostic criteria, and follow-up periods. Moreover, although most of the pediatric studies used Lactobacillus rhamnosus GG alone or in combination with other probiotics, some studies administered other bacterial strains, alone or in combination. The dosages and duration of prophylactic treatment were frequently very different among the studies. Consequently, the effect of different strains of probiotics and the best dosage and schedule of administration were not assessed.

A second meta-analysis, including studies carried out until July 2014, examined 13 randomized controlled trials, globally enrolling 3720 individuals among children, adults, and elderly people [103]. As in the previous meta-analysis, probiotics were found to be better than placebo when some primary endpoints of the trials were considered. The number of participants experiencing upper RTIs during the study period was lower among treated individuals than among controls (at least one episode: OR 0.53; 95% CI 0.37–0.76, *p* < 0.001; at least three episodes: OR 0.53; 95% CI 0.36–0.80, *p* = 0.002). Moreover, compared with controls, treated children showed a lower mean duration of each infectious episode (MD −1.89; 95% CI from −2.03 to −1.75, *p* < 0.001), lower rates of antibiotic prescription (OR 0.65; 95% CI from 0.45 to 0.94), and fewer disease-associated school absences (OR 0.10; 95% CI from 0.02 to 0.47). However, probiotics had no effect on the rate ratio of episodes of acute upper RTIs (rate ratio (RR) 0.83; 95% CI from 0.66 to 1.05, *p* = 0.12) or on the number and type of adverse events (OR 0.88; 95% CI from 0.65 to 1.19, *p* = 0.40). Nevertheless, considering the study characteristics and the high level of heterogeneity among them, the evidence level of results was considered of low quality, and the real efficacy of probiotics was not clearly demonstrated. Moreover, no relevant information on differences among the various probiotics was reported.

Finally, a third meta-analysis included 21 randomized clinical trials published up until November 2015, enrolling children and adolescents. In this case, quite different results were reported [104]. When all the studies were considered together, it was evidenced that the number of respiratory episodes during the follow-up period was quite similar in the group of children given probiotics and in those receiving placebo. However, if results due to different probiotics were analyzed, it was shown that contrarily to other probiotics that had no effect, the administration of Lactobacillus casei rhamnosus LCA was associated with a reduced risk of RTI development (RR 0.38; credibility interval 0.19–0.45). Even these results could be debated, as LCA was used in only three trials enrolling 1731 participants with very different ages who received the probiotic for different times and at different dosages.

## 5. Vitamins

Some studies have measured the clinical relevance of vitamin A (VA) and C (VC) supplementation for the prophylactic treatment of RTIs. The results of studies carried out in healthy children or in children at risk are few and conflicting [105,106]. No reliable VA or VC data are available for the prevention of recurrences in RTI-prone children. Consequently, neither VA nor VC have a role in this regard.

On the contrary, recent studies have shown that in addition to the known activity on bone mineralization and growth, vitamin D (VD) exerts several other actions. Among these, the modulation of both innate and adaptive immunity with increased efficiency of several infection protection mechanisms is one of the most important [107,108,109,110,111,112,113,114]. Moreover, several observational studies have reported an independent association between susceptibility to RTIs and low VD 25(OH) serum levels [115,116]. Both of these factors have prompted numerous randomized controlled trials to determine whether VD supplementation could reduce the risk of acute RTIs. Unfortunately, as already reported for probiotics, most of the studies have simply evaluated the impact of VD on the incidence of RTIs in the general pediatric population. Only in a few cases were children with a history of recurrent RTIs enrolled, and the results were conflicting. A study evidenced that the administration of oral VD 1000 IU/day for 4 months in children with recurrent AOM could significantly reduce the incidence of new AOM episodes in the 6 months following the beginning of supplementation [117]. The number of children experiencing ≥1 AOM episode during the study period was significantly lower in the treatment group (*p* = 0.03). However, the likelihood of new AOMs was significantly reduced only in patients with VD hypovitaminosis at baseline (<30 ng/mL) and in those with a history of uncomplicated AOMs. Children who had suffered from AOM with repeated tympanic membrane perforation gained no advantage from VD supplementation, even if they had low VD serum concentrations at baseline. Negative results were also collected in children with recurrent wheezing, which is a condition that, particularly in the youngest children, is frequently associated with a viral RTI, while a series of randomized controlled trials showed inconsistent results on the role of VD in reducing the risk of asthma attacks and in asthma symptom control [118]. 

However, if the poor number of previous studies does not allow us to include VD administration among the measures that can be used to reduce new episodes of disease in children with recurrent RTIs, recently collected data seem to indicate that VD truly could have potential efficacy in this regard and that new studies are mandatory to definitively assess when and how VD could be useful. Starting from the evidence that meta-analyses measuring the impact of VD on acute RTI development in children in the past have led to contrasting results, probably due to an inadequate selection of the studies [119,120,121,122,123], a new meta-analysis carried out with different modalities was performed [124]. Only prospective, randomized, double-blind, placebo-controlled trials of VD supplementation of any duration were considered. Moreover, to reduce the negative influence of the heterogeneity of studies on the results of the meta-analysis, a series of subgroup evaluations was performed. Among the other variables, participant characteristics, including the type of respiratory disease, age, and body mass index; dosing regimens; and baseline VD levels were considered. Acute RTIs revealed that VD supplementation had a strong protective effect on patients with VD baseline levels <10 ng/mL (adjusted OR (aOR) 0.58, 95% CI from 0.40 to 0.82; *p* = 0.002), but no effect was observed among those with a baseline VD concentration of ≥10 ng/mL (aOR 0.89, 95% CI from 0.77 to 1.04; *p* = 0.15). The effect was particularly evident in children aged 1.1-15.9 years: proportion with ≥1 acute RTI (34.3% vs. 47.0%; aOR 0.60, 95% CI 0.46–0.77; *p* < 0.001). Finally, whereas daily VD doses were effective, bolus-dose VD supplementation did not offer any protection against acute RTI even when administered to patients with hypovitaminosis (aOR 0.82, 95% CI 0.51 to 1.33; *p* = 0.43). All these findings suggest that VD supplementation, at least in children with VD deficiency, could play a role in children with recurrent RTIs. However, only further methodologically adequate studies carried out in children with documented recurrent RTIs that are capable of defining which is the lowest minimum VD serum level that is strongly associated with an increased risk of RTI development, which is the most effective dosage and schedule of administration and how long treatment must be administered, can solve the problem.

## 6. Complementary and Alternative Medicines

Complementary and alternative medicine (CAM) includes several different treatment measures that fall outside the realm of conventional medicine. Herbal medicines, bee products, and homeopathy are among those most frequently used to prevent and treat respiratory infections in children [125,126,127]. In some cases, such as in the case of herbal medicines and bee products, some assumptions based on biological data lead to the hypothesis that these products can be of benefit. In other cases, such as in the case of homeopathy, no real scientific assumption justifies their use [127]. Echinacea and Pelargonium sidoides are the most widely prescribed herbal medicines. Echinacea contains variable amounts of ingredients with pharmacological activity, such as polysaccharides, chicory acid glycosides, essential oils, oxyacetylene, and alkyl amides. 

In vitro and experimental studies have shown that Echinacea stimulates the macrophage production of cytokines (i.e., tumor necrosis factor-α, interleukin (IL)-10, IL-6, and IL-1) and exerts bactericidal and antiviral activity [127,128,129]. Pelargonium sidoides has documented pharmacological activities, including antiviral and antibacterial action, as well as immune-modulatory capabilities, which are mainly evidenced by the activation of macrophages and the increase in the production of nitric oxide [130]. However, despite these biological advantages, no clear evidence of efficacy for any herbal medicines in the prevention of RTIs in humans is available. This lack of clear evidence is highlighted in the conclusions of a systematic review and meta-analysis [131] of all the randomized controlled trials carried out in children and adolescents up until February 2015. These data explain why herbal products cannot be recommended for the prevention of recurrences in RTI-prone children.

Two honeybee products, propolis and royal jelly, are included among complementary and alternative medicines. Both contain ingredients that have antioxidant, immunomodulatory, antibacterial, antiviral, and anti-inflammatory properties [131,132,133]. Very few data regarding the use of these compounds in randomized clinical trials in children with recurrent RTIs are available. Positive results were evidenced when a suspension of propolis and zinc was used to prevent AOM in 122 children aged 1-5 years with a documented history of recurrent AOM [134]. In this case, a prospective, blindly randomized trial was carried out. In the 3-month treatment period, the incidence of AOM was significantly lower in treated children than in controls (50.8% vs. 70.5%; *p* = 0.04). However, the lack of well-conducted studies including RTI-prone children receiving honeybee products alone and children suffering from RTIs other than AOM does not allow us to draw definitive conclusions for the use of these products in clinical practice. Moreover, particular attention must be paid to the evaluation of the safety and tolerability of honeybee products because of the possible risk of allergic reaction and sensitization due to the pollen content. In conclusion, no recommendation for the use of honeybee products is presently possible.

Totally negative conclusions can be drawn for homeopathy. A recent Cochrane review that analyzed eight randomized controlled trials comparing oral homeopathy medicinal products with identical placebo or self-selected conventional treatments to prevent or treat acute RTIs in children aged 0 to 16 years supports this [29]. When the four studies regarding prevention were pooled, it was found that homeopathic medicinal products conferred no preventive effect on acute RTIs (OR 1.14, 95% CI 0.83–1.57) and were not associated with a reduction of the need for antibiotic usage (OR 0.79, 95% CI 0.35 to 1.76). An evaluation of adverse events, hospitalization rates and length of stay, days off school or work for parents, and quality of life was not possible. Moreover, when the only study enrolling children with recurrent RTIs was considered, the results were not substantially different, as prescribed homoeopathic medicines seemed to add little to a careful counseling in reducing the daily burden of symptoms, the use of antibiotics, and the need for adenoidectomy and tonsillectomy. In both children with and without homeopathic medicines, the use of antibiotics was reduced compared with that in the year before entering the trial (from 73 to 33 in the treatment group and from 69 to 43 in the placebo group). The proportion of children in the treatment group having adenoidectomies was lower in the treatment group (16%, 8/50) than in the placebo group (21%, 9/42). The proportion having tonsillectomies was the same in both groups (5%). However, the reliability of these very modest results is affected by the very low quality of all the studies regarding homeopathic medicine use.

## 7. Conclusions

Despite otherwise healthy children with recurrent RTIs being a sizable part of the pediatric population and recurrent RTIs having a significant impact on sick children, their family, society, and the health system, few convincing measures for reducing the frequency and clinical relevance of recurrent respiratory episodes in RTI-prone children have been developed. Table 1 summarizes the available evidence and priorities for future research. Among the most recently suggested measures, immunotherapy is attractive, but only for OM-85 is there a sufficient number of well-conducted clinical trials confirming efficacy in RTIs prevention with an adequate safety profile. In the case of probiotics, it is not clear which bacteria can offer the best results and which dosage and schedule of administration are the most effective. The problems of dosage and the schedule of administration are not solved also for VD, despite some interesting efficacy results. While we wait for new knowledge on the detection of biomarkers, including microbiome taxa, which are able to support the identification of the best responder profile and a precise host-tailored medicine, the elimination or reduction as much as possible of the environmental factors that favor RTIs, vaccination when available and/or indicated, and the systematic application of the traditional methods for infection prevention, such as hand washing, remain the best measures to prevent recurrent infections in RTI-prone children.

## Figures and Tables

**Table 1:** Consensus statements on products used for prevention of respiratory tract infections (RTIs) in RTI-prone children.

Product	Main Data	Main Limitations	Consensus Statement and Suggestions for Future Research
Pidotimod	Positive influence on innate and adaptive immunity in vitro, efficacy in prevention of RTIs in RTI-prone children, duration and severity of respiratory symptoms, antibiotic use, good safety profile.	Licensed for children ≥3 yrs, to be given 2 hrs before or after meals, available only in few countries, few studies available with sufficient details on randomization method and using blind approach, heterogeneity in dosages and schedule of administration.	Pidotimod could play a role in prevention of respiratory recurrences in RTI-prone children ≥3 yrs old, although further randomized, double-blind studies are needed to confirm population that could have advantages and to define the dosages and schedule of administration.
OM-85	Positive influence on innate and adaptive immunity in vitro, downregulation of inflammatory state, efficacy in prevention of RTIs in RTI-prone children, duration and severity of respiratory symptoms, antibiotic use, days of absence from day-care of children and working days lost by parents, efficacy in children with recurrent wheezing and asthma, excellent safety profile.	Absence of biomarkers able to predict the best responder profile and a precise-host tailored medicine.	OM-85 should be recommended for prevention of respiratory recurrences in RTI-prone children ≥6 months old, although further studies on detection of biomarkers able to support the identification of best responder profile and a precise-host tailored medicine are needed.
Ribomunyl	Modulation of innate and adaptive immunity in vitro, some clinical evidence in reduction of RTI and antibiotic courses.	Availability of few studies with enrolment of a relatively low number of children. Not available on the market worldwide anymore.	Ribomunyl cannot be recommended for the prevention of recurrences in RTI-prone children.
PBML and LW50020	Stimulation of innate and adaptive immunity in vitro.	Few clinical evidences.	PBML and LW50020 cannot be recommended for the prevention of recurrences in RTI-prone children.
B-glucans	Enhancement of activity of innate and adaptive immunity in vitro.	Contrasting results in efficacy against respiratory recurrences, good safety and tolerability profile.	B-glucans cannot be recommended for the prevention of recurrences in RTI-prone children.
Probiotics	Modulation of innate and adaptive immunity in vitro, main data on *Lactobacillus* spp. and *Bifidobacterium* spp. that in some studies reduced episodes of upper RTI, antibiotic use and school absences.	Very few data on RTI-prone children, heterogeneity in type of probiotic tested, dose and duration of administration.	Probiotics cannot be recommended for the prevention of recurrences in RTI-prone children.
Vitamins	Vitamin A and vitamin C: No reliable data on vitamin A and vitamin C Vitamin D: Modulation of innate and adaptive immunity in vitro, safe protection against acute RTIs, with major benefits in very deficient individuals and those not receiving bolus doses.	Vitamin A and vitamin C: No evidence for the prevention of RTIs in children. Vitamin D: Few data on RTI-prone children.	Vitamin A and vitamin C cannot be recommended for the prevention of recurrences in RTI-prone children. Vitamin D could play a role in children with recurrent RTIs, although further methodologically adequate studies in RTI-prone children are needed to clarify the lowest minimum vitamin D serum level associated with an increased risk of RTIs, the most effective dosage, schedule of administration and duration of treatment.
*Echinacea*	Stimulation of macrophage with production of cytokines as well as antiviral and antibacterial action in vitro.	No evidence for the prevention of RTIs in humans.	Echinacea cannot be recommended for the prevention of recurrences in RTI-prone children.
Honeybee products (propolis and royal jelly).	Antioxidant, immunomodulatory, antibacterial, antiviral and anti-inflammatory properties in vitro.	Effect in only one study on recurrent acute otitis media; absence of well-conducted studies including RTI-prone children suffering from RTIs other than otitis.	Honeybee cannot be recommended for the prevention of recurrences in RTI-prone children.
